# CAR T-cell therapy in pediatric brain tumors: a narrative review with comparative analysis of clinical trial eligibility criteria

**DOI:** 10.3389/fonc.2026.1869826

**Published:** 2026-07-24

**Authors:** Akshaya Srinivasan, Angeline Abraham, Aliya I. Modak, Anugrah Pradeep, Nisrine L. Nimachwala, Akshit V. Chemmancheri, Aarifa Banu, Afnan A. Azad, Ashly Abraham, Lika Khorbaladze

**Affiliations:** 1 Tbilisi State Medical University, Tbilisi, Georgia; 2 Ivane Javakhishvili Tbilisi State University, Tbilisi, Georgia; 3 New Vision University, Tbilisi, Georgia; 4 Georgian National University SEU, Tbilisi, Georgia; 5 Iashvili Children’s Hospital, Tbilisi, Georgia

**Keywords:** antigen escape, B7-H3, CAR T-cell therapy, CNS tumors, GD2, immunotherapy, neurotoxicity, pediatric brain tumors

## Abstract

Pediatric brain tumors are the leading cause of cancer-related mortality in children, and current standard therapies like surgery, radiotherapy, and chemotherapy offer limited survival benefits and significant long-term morbidity. Chimeric antigen receptor (CAR) T-cell therapy is a transformative treatment for hematologic malignancies and is now being explored for pediatric brain tumors. This review summarizes the latest advances, preclinical and clinical findings, challenges of CAR T-cell therapy, and future directions in pediatric neuro-oncology. 18 studies that met the eligibility criteria were selected, consisting of preclinical models, early-phase clinical trials, and translational studies. A registry search of central nervous system (CNS) tumor trials from Clinicaltrials.gov, ISRCTN, and ANZCTR identified 12 active or completed interventional trials of CAR T-cell therapy in patients with CNS tumors, their eligibility criteria and parameters were compared. Preclinical studies consistently demonstrate that CAR T-cells targeting antigens such as B7-H3, GD2, HER2, IL13Rα2, and EphA2 can induce robust and specific tumor regression in models of medulloblastoma, diffuse intrinsic pontine glioma (DIPG), ependymoma, and high-grade gliomas. On the other hand, B7-H3 is a pan-pediatric target due to its high expression in multiple CNS tumors, including medulloblastoma, ependymoma, and glioma, whereas GD2 is highly relevant for H3K27M-mutant diffuse midline gliomas. Early-phase clinical trials confirm that CAR T-cells can traffic to CNS tumors, infiltrate tumor tissue, and mediate tumor regression. The ICV B7-H3 phase 1 trial in DIPG achieved noteworthy results, with a median survival of 19.8 months across 21 patients and 3 patients surviving more than 40 months. GD2-CAR T-cell therapy in H3K27 M-mutant gliomas showed partial clinical responses, with neurotoxicity and encephalopathy observed, whereas the HER2-targeted locoregional therapy showed no dose-limiting toxicities. Future interventions such as multi-antigen targeting, combinatorial CAR designs, and enhanced cytokine signaling are being developed to improve efficacy and safety. A comparison of 12 registered pediatric CAR T-cell trials showed heterogeneity in eligibility criteria, including age ranges, performance status thresholds, H3K27M mutation requirements, and geographic concentration bias. CAR T-cell therapy holds significant promise for improving outcomes in pediatric brain tumors, but its clinical translation is challenged by tumor heterogeneity, antigen escape, neurotoxicity, and the immunosuppressive tumor microenvironment.

## Introduction

Pediatric brain tumors are the leading cause of cancer-related mortality among children with solid malignancies and account for a substantial proportion of childhood cancers (1–3). Although there has been progress with some subtypes, the outcomes for aggressive and anatomically challenging tumors are still poor. For example, diffuse intrinsic pontine glioma (DIPG) and other diffuse midline gliomas grow in an infiltrative manner, limiting surgical options, and often result in survival measured in months (1, 4). Standard treatments like surgery, radiotherapy, and chemotherapy can lead to long-term cognitive, endocrine, and functional side effects during crucial developmental stages (3). The high mortality rate and long-term complications highlight the need for treatments that are more effective and more targeted.

**Figure 1:**
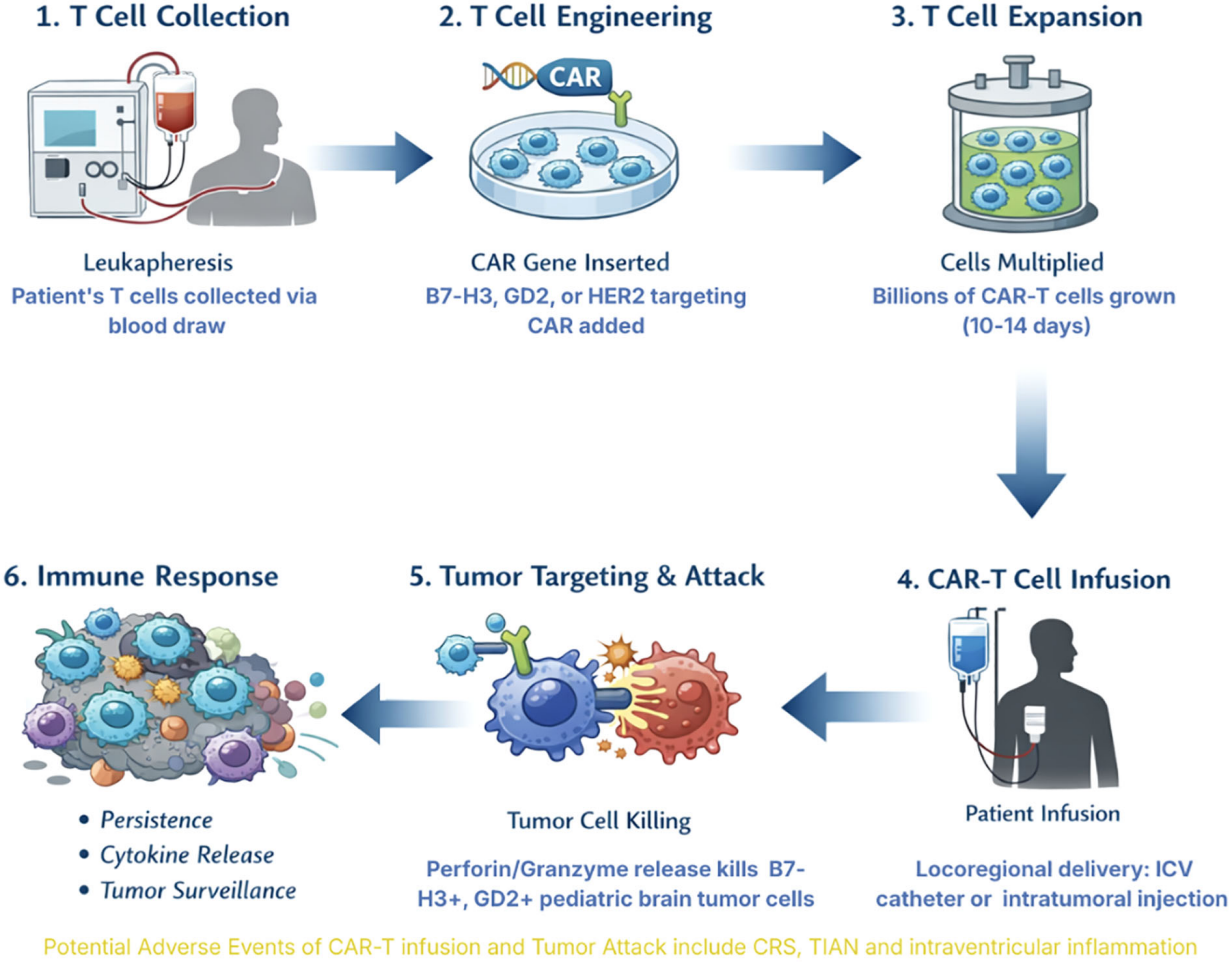
CAR T-cell therapy process for pediatric CNS tumors. (1) Patient T cells are collected, (2) engineered to express tumor-specific CARs targeting B7-H3/GD2/HER2, (3) expanded ex vivo, and (4) delivered locoregionally via ICV catheter or intratumoral injection. (5) CAR T-cells recognize and lyse antigen-positive tumor cells through perforin/granzyme-mediated cytotoxicity and (6) recruit endogenous immunity for sustained tumor control. Potential adverse events associated with CAR T-cell infusion and tumor targeting include cytokine release syndrome (CRS), tumor inflammation-associated neurotoxicity (TIAN), and intraventricular inflammation. B7-H3, B7 homolog 3 protein; CAR, chimeric antigen receptor; CNS, central nervous system; CRS, cytokine release syndrome; GD2, disialoganglioside 2; HER2, human epidermal growth factor receptor 2; ICV, intracerebroventricular; TIAN, tumor inflammation-associated neurotoxicity.

Furthermore, immunotherapy has transformed cancer treatment across different malignancies, including CAR T-cell therapy, particularly for hematological cancers in patients who are resistant or refractory (2, 6). In CAR T-cell therapy, autologous or allogeneic T cells are genetically modified to express chimeric antigen receptors that recognize tumor antigens, thereby enabling them to mediate MHC-independent cytolysis (2, 3, 7) (**Figure 1**). Although applying the same mechanism in the treatment of solid tumors, including brain tumors in children, may prove to be difficult, the underlying scientific rationale remains compelling.

A primary determinant of this approach is the identification of tumor-associated antigens that are highly expressed on malignant cells and minimally expressed in normal brain tissue. Key targets focused on in pediatric neuro-oncology include B7-H3 (CD276), GD2, HER2, IL13Rα2, and EphA2 (5–9). Many of these antigens offer opportunities for broader applicability, while others may enable biologically guided selection of patient subgroups most likely to benefit. However, there are additional complications with the CNS: the blood-brain barrier (BBB) limits T-cell entry after intravenous infusion, intracranial space limitations predispose to inflammation and edema, and tumoral heterogeneity promotes antigen escape and immune evasion (2, 4, 10).

This review focuses on the important preclinical and early clinical studies investigating the use of CAR T-cell treatment for pediatric brain tumors, with particular emphasis on target selection, modes of delivery, signs of effectiveness, safety trends, and key biological and logistical challenges that need to be addressed before sustained benefits can be achieved. This review also offers a structured comparative analysis of the eligibility criteria used across ongoing CAR T-cell trials in children with CNS malignancies, identifying patterns of heterogeneity with implications for patient access and future trial design.

## Methodology

A systematic literature review in PubMed, Embase, and Web of Science identified pertinent literature up to 2025, using combinations of Boolean operators with combinations of keywords, such as “CAR T-cell therapy” AND “pediatric” OR “childhood” AND “brain tumor” OR “CNS tumor”, along with additional terms such as B7-H3, GD2, HER2, IL13Rα2, EphA2, DIPG, diffuse midline glioma, medulloblastoma, and ependymoma.

Inclusion Criteria: Studies were included if they reported original data in English, on pediatric CNS tumors or on representative pediatric CNS models, addressing target antigen expression, CAR construct design, delivery route, safety profile, or therapeutic efficacy. Both preclinical (cell-based or animal model) and clinical studies were included.

Exclusion Criteria: Studies were excluded for duplicate records, non-CNS tumor populations or adult-only cohorts (“wrong population”), insufficient original outcome data (e.g., editorials, commentaries, or abstracts without extractable results) or publication in a language other than English.

**Figure 2:**
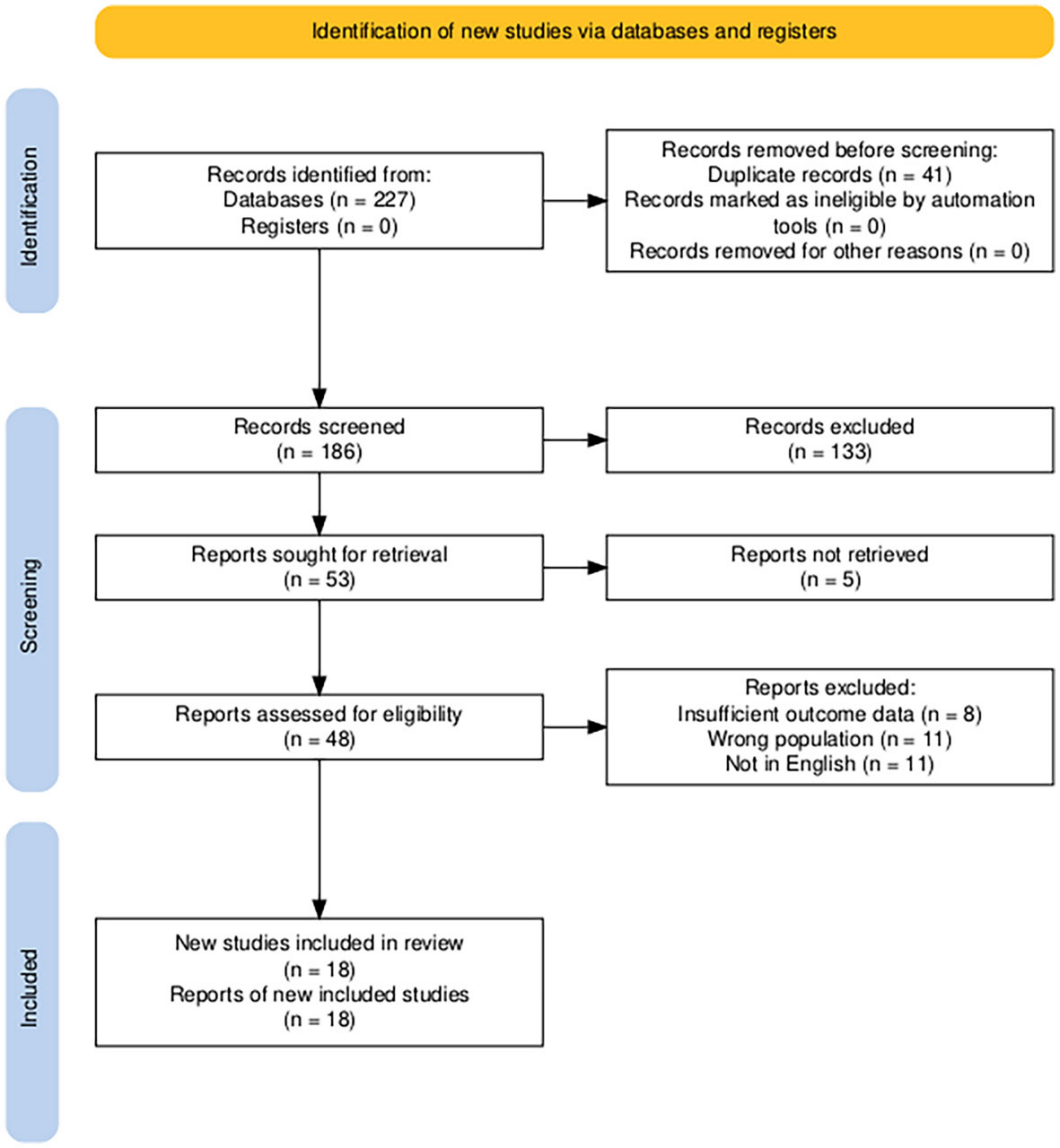
Study identification and selection flow diagram. Records identified through database searching (n=227) were screened after removal of duplicates (n=41), yielding 186 records for title/abstract screening; 133 were excluded at this stage. Of the 53 reports sought for full-text retrieval, 5 were not retrieved, leaving 48 assessed for eligibility. Thirty reports were excluded at full-text review (insufficient outcome data, n=8; wrong population, n=11; not in English, n=11), resulting in 18 studies included in the narrative synthesis.

Study Selection: Database searches identified 227 records, from which none came from registers. After removing 41 duplicate records (no records were excluded by automation tools or for other reasons), 186 records underwent title/abstract screening, of which 133 were excluded as they did not meet the inclusion criteria. The remaining 53 reports were sought for full-text retrieval, from which 5 could not be retrieved, leaving 48 assessed for eligibility. Of these, 30 were excluded (8 for insufficient outcome data, 11 for wrong population, 11 not in English), finally producing 18 studies included in the narrative synthesis (**Table 1**; **Figure 2**).

**Table 1:** Summary of selected studies on CAR T-cell therapy in pediatric central nervous system tumors, including preclinical, clinical, and review evidence on target antigens, therapeutic efficacy, and translational challenges.

Authors	Study design	Key findings
Majzner et al. (2019) (8)	Original research (preclinical study)	B7-H3 CAR T-cells have demonstrated a strong ability to shrink tumors and eliminate xenografts while maintaining low off-tumor toxicity in models of pediatric solid tumors and brain tumors.
Vitanza et al. (2025) (5)	Original research (Phase 1 clinical trial)	Administering B7-H3 CAR T-cell infusions intracerebroventricularly was associated with minimal toxicity and achieved a median overall survival of ~19.8 months in DIPG.
Mount et al. (2018) (4)	Original research (preclinical study)	GD2 CAR T-cells selectively targeted H3K27M-mutant glioma cells, leading to tumor clearance and significantly prolonged survival in orthotopic models.
Ronsley et al. (2024) (1)	Review	Current research on CAR T-cell therapy demonstrates feasibility and early signs of effectiveness in pediatric CNS tumors, but biological and delivery barriers remain, affecting its durability.
Vitanza et al. (2021) (12)	Original research (early-phase clinical study)	Locoregional HER2 CAR T-cell delivery was well tolerated with no dose-limiting toxicities reported and showed evidence of disease stabilization and partial responses in some patients.
Wang et al. (2023) (13)	Original research (interim analysis)	IL13Rα2-targeted CAR T-cell therapy led to an increase of endogenous CD8+ T cells in CSF, but CAR T-cell persistence in the TME remained limited.
Rao et al. (2022) (2)	Review	Some of the major barriers against CAR T-cell therapy include antigen heterogeneity, immunosuppressive TME, and limited persistence, ultimately impacting its efficacy in pediatric brain tumors.
Yaacoub et al. (2025) (3)	Review	The efficacy of CAR T-cell therapy in pediatric brain malignancies is limited by T-cell exhaustion, limited persistence within the TME, and poor penetration of the BBB.
Haydar et al. (2021) (9)	Original research (translational study)	B7-H3 was consistently and highly expressed across multiple pediatric brain tumor types, making it a potential broad therapeutic target.
Patterson et al. (2020) (7)	Review	CAR T-cell therapy appears promising for pediatric brain tumor treatment, but challenges in antigen selection, delivery, and safety limit its current effectiveness in clinical settings.
Burns et al. (2022) (10)	Review	For effective clinical translation of CAR T-cells, overcoming tumor heterogeneity, immune evasion, and delivery limitations specific to CNS tumors is required.
Vitanza et al. (2023) (16)	Original research (clinical procedural study)	Intracranial catheter-based CAR T-cell delivery was safe across multiple administrations, with no CSF infections or major device-related complications.
Timpanaro et al. (2023) (17)	Review	CAR T-cell delivery for CNS tumors requires locoregional administration as systemic delivery is challenged by the Blood–brain barrier and tumor infiltration.
Thomas et al. (2021) (14)	Review	Preclinical and early clinical data support CAR T-cell as a promising therapy, but optimization of targets and delivery is essential for efficacy.
Ferreras et al. (2021) (18)	Review	The key drivers of treatment resistance in pediatric brain tumors are tumor antigen escape, heterogeneity, and immunosuppressive microenvironment.
Wu et al. (2021) (11)	Review	Immunosuppressive signaling pathways and interactions within the TME significantly impair the functioning and persistence of CAR T cells.
Huang et al. (2020) (6)	Review	Clinical gaps in CAR T-cell therapy, including targeting, improved persistence, and safe administration, need to be addressed for its proper use.
Majzner et al. (2022) (15)	Original research (clinical study)	GD2 CAR T-cell therapy produced favorable clinical responses, including tumor regression and neurological improvement, with manageable but notable neurotoxicity.

B7-H3, B7 homolog 3 protein; BBB, blood-brain barrier; CAR T-cell, chimeric antigen receptor T-cell; CNS, central nervous system; CSF, cerebrospinal fluid; DIPG, diffuse intrinsic pontine glioma; DLTs, dose-limiting toxicities; GD2, disialoganglioside 2; H3K27M, histone H3 lysine-27-to-methionine mutation; HER2, human epidermal growth factor receptor 2; ICV, intracerebroventricular; IL13Rα2, interleukin-13 receptor alpha 2; OS, overall survival; TME, tumor microenvironment.

In parallel with the literature review, a separate search was conducted in April 2025 in ClinicalTrials.gov, the ISRCTN registry, and ANZCTR to identify active interventional trials of CAR T-cell therapies in pediatric patients with primary CNS tumors. Search terms included CAR T-cell, chimeric antigen receptor, pediatric, brain tumor, glioma, DIPG, diffuse midline glioma, and CNS; any trials restricted to blood cancers were excluded. Critical parameters were recorded in each qualified trial: the trial identifier, antigen of interest, eligible tumor types, patient age range, performance status requirement, H3K27M mutation status requirement, route of administration, previous therapy requirement, trial status, and the sponsor. Overall, twelve trials were advanced to the eligibility evaluation and are summarized in **Table 2**.

**Table 2:** Summary of registered clinical trials evaluating CAR T-cell therapy in pediatric central nervous system tumors, including target antigens, eligibility criteria, routes of administration, and trial status (19, 20).

No	Trial ID	CAR target	Tumor scope	Age range	Perf. status (scale)	H3K27M	Route	Prior Rx	Status	Country
1	NCT05768880	B7-H3+EGFR806+HER2+IL13	DIPG/DMG/CNS	1–26 yrs	≥60 (Lansky/Karnofsky)	Not required	ICV	No	Active	USA
2	NCT05298995	GD2	HGG/MB/DMG/ DIPG	6m–30 yrs	≥60 (Lansky/Karnofsky)	Not required	IV	No	Recruiting	Italy
3	NCT03638167	EGFR+	R/R EGFR+ CNS	15–26 yrs	≥60 (Karnofsky)	Not required	IV	No	Completed	USA
4	NCT07087002	GPC2	Medulloblastoma	12m–30 yrs	Not stated	Not required	ICV	No	Recruiting	USA
5	NCT03500991	HER2+EGFRt	HER2+ R/R CNS	1–26 yrs	≥60 (Lansky/Karnofsky)	Not required	IV	No	Active	USA
6	NCT04185038	B7-H3+EGFRt	DIPG/DMG/ R/R CNS	1–26 yrs	≥60 (Lansky/Karnofsky)	Not required	IV	No	Recruiting	USA
7	NCT06946680	CD70	HGG (WHO III–IV)	4–18 yrs	>70 (Lansky/Karnofsky)	Not required	ICV	No	Active	USA
8	NCT07390539	B7-H3	Embryonal/ Ependymoma	2–21 yrs	≥60 (Lansky/Karnofsky)	Not required	ICV ± intratumoral	Yes	Not yet recruiting	USA
9	NCT04099797	GD2	DMG/HGG/DIPG/MB	12m–22 yrs	≥50 (Lansky/Karnofsky)	Conditional	IV+ICV	Yes	Recruiting	USA
10	NCT04510051	IL13Rα2	R/R brain tumors	4–25 yrs	≥60 (Lansky/Karnofsky)	Not required	ICV	Yes	Recruiting	USA
11	ISRCTN15356845	IL-15/06B5	Diffuse HGG	2–16 yrs	≥40 (Lansky)	Not required	IV→ICV	Yes	Recruiting	UK
12	ACTRN12622000675729	GD2	DMG/DIPG	≤21 yrs	≥50 (Lansky/Karnofsky)	Required	IV→ICV	Yes	Recruiting	Australia

B7-H3, B7 homolog 3 protein; CAR, chimeric antigen receptor; CD70, cluster of differentiation 70; CNS, central nervous system; DIPG, diffuse intrinsic pontine glioma; DMG, diffuse midline glioma; EGFR, epidermal growth factor receptor; EGFR806, EGFR epitope 806; EGFRt, truncated EGFR (safety marker); GD2, disialoganglioside 2; GPC2, glypican-2; H3K27M, histone H3 lysine-27-to-methionine mutation; HER2, human epidermal growth factor receptor 2; HGG, high-grade glioma; ICV, intracerebroventricular; IL-15/06B5, interleukin-15 co-stimulated CAR construct; IL13, interleukin-13; IL13Rα2, interleukin-13 receptor alpha 2; IV, intravenous; m, months; MB, medulloblastoma; Perf., performance status; Prior Rx, prior treatment required; R/R, relapsed/refractory; WHO, World Health Organization; yrs, years.

*Efficacy data (ORR, PFS, OS) and major toxicity (DLTs) are not reported for ongoing trials, as the majority of trials remain active or recruiting and have not yet published outcome data. For the one completed trial (NCT03638167, EGFR-targeting), available efficacy and safety data are discussed in the manuscript text. Readers are directed to the original publications and ClinicalTrials.gov for updated results as they become available.

## Results

### Target antigens in pediatric brain tumors

#### B7-H3 (CD276)

B7-H3 is one of the most frequently expressed cell-surface antigens in pediatric solid tumors, including several pediatric CNS tumors (8, 9). In early preclinical research, B7-H3-targeting CAR T-cells demonstrated strong antitumor activity and eliminated xenograft tumors in pediatric solid-tumor models, thereby establishing this antigen as a viable target for CAR-based therapy (8). Antigen profiling and translational studies showed that B7-H3 is broadly expressed in medulloblastomas, ependymomas, and gliomas, which led to the concept of B7-H3 as a “pan-pediatric” target in CNS tumors (8, 9). The first-in-human phase 1 trial of intracerebroventricular (ICV) B7-H3-targeting CAR T-cells in DIPG further validated B7-H3 as a clinically actionable target, demonstrating that high-density expression across tumor subtypes supports multi-cycle targeted delivery (5).

Pediatric tumors can be molecularly heterogeneous. Using a target that is both frequently expressed and present at high density can reduce the risk of therapy failure due to the antigen not being expressed at baseline. However, heterogeneity within the tumor itself can still allow escape (2, 10, 11).

#### GD2

GD2 is a widely recognized disialoganglioside associated with diffuse midline gliomas, including H3K27M-altered tumors. In a landmark preclinical study, GD2-targeted CAR T-cells showed strong antitumor activity in H3K27M-mutant diffuse midline glioma models, supporting GD2 as a target for DIPG-focused immunotherapy development (4). GD2-CAR approaches have progressed to pediatric CNS clinical testing, with early-phase data—including from a published Nature trial—showing viability, manageable toxicity, and initial clinical activity, such as partial responses or disease stabilization, in children with diffuse midline gliomas and high-grade gliomas (1, 4, 15). As a result, GD2 is noteworthy for both the relative maturity of its clinical translation and the strength of its preclinical validation in lethal midline disease.

#### HER2

HER2 expression varies more widely in pediatric brain tumors than B7-H3 does, but is still significant in some glioma and medulloblastoma subtypes (7, 9). Locoregional infusion of HER2-specific CAR T-cells in children and young adults with recurrent or refractory CNS tumors has demonstrated feasibility and tolerability, with evidence of initial clinical activity in some patients (12). Careful dosing and safety measures are necessary given historical concerns about off-tumor toxicity for HER2-targeted therapies. In the pediatric context, HER2 is considered alongside other antigens as a potential target for selected cases or multi-antigen strategies (1, 7).

#### IL13Rα2 and EphA2

IL13Rα2 has been identified as a glioma-associated antigen with limited expression in normal tissues. An interim analysis of locoregional IL13Rα2-targeting CAR T-cell delivery demonstrated that intraventricular administration in pediatric CNS tumor patients was safe and feasible, with evidence of clonal expansion of endogenous CD8+ T cells in the cerebrospinal fluid (CSF) and radiographic benefit in some patients (13). EphA2 is another candidate antigen implicated in tumor invasiveness and malignant signaling. Early-stage evidence indicates tumor specificity and relevance for engineered cellular therapies; however, the development of this antigen is less advanced than that of B7-H3 or GD2 in pediatric CNS disease (9, 10).

### Preclinical efficacy

Across preclinical studies, CAR T-cell therapy has exhibited proof-of-principle antitumor activity in pediatric CNS tumor models. B7-H3 CAR T-cells have shown strong tumor control and xenograft eradication in pediatric solid tumor and brain tumor models, providing some of the strongest preclinical validation for a broadly expressed pediatric target (8, 9, 14). Similarly, GD2-directed CAR T-cells have demonstrated robust efficacy against tumor progression and extended survival in diffuse midline gliomas, an accomplishment given the historically poor prognosis of DIPG due to a lack of systemic treatment options (4). HER2-directed CAR T-cells have inhibited tumor growth in key xenograft models, illustrating their potential for selected tumors with sufficient HER2 expression (7, 12).

In addition to target selection, preclinical research increasingly focuses on engineering strategies to overcome the hostile CNS tumor microenvironment (TME), characterized by immunosuppressive cytokines, inhibitory ligands, and metabolic constraints that limit T-cell persistence and function (2, 4). Armored CAR models, including CAR T-cells engineered for enhanced persistence or resistance to suppression, represent a significant area of innovation. The development of cytokine support mechanisms and receptor-altering techniques for sustained efficacy against pediatric solid and brain tumors marks a shift from the early cytotoxic generation to a new approach that emphasizes persistence and resistance (2, 3, 5). These studies highlight the biological feasibility of using CAR T-cells for pediatric central nervous system tumors and underscore the importance of considering alternative approaches beyond current hematological CAR designs.

### Clinical translation

Clinical practice remains preliminary and involves small patient groups, yet the outcomes have been promising enough to warrant further trial expansion and optimization. The phase 1 trial of GD2-CAR T-cell therapy in children with H3K27M-Mutant diffuse midline gliomas showed relatively high toxicity rates, but adverse events were generally manageable, with early signs of clinical activity, including partial responses or disease stabilization (1, 4, 15). Similarly, a preliminary trial of B7-H3 ICV therapy in patients with DIPG demonstrated a median overall survival of 19.8 months from diagnosis, across 253 doses administered to 21 patients—significantly exceeding that reported in historical controls—with three patients surviving beyond 40 months (5).

Locoregional delivery of HER2- CAR T-cell therapy is feasible, with no dose-limiting toxicities, in a pediatric and young-adult cohort with recurrent or refractory CNS tumors, providing valuable lessons on safety and tolerability (12). The clinical procedure for locoregional delivery, including catheter placement and safety monitoring across 307 intracranial doses at one institution, has been characterized in detail and found to be manageable with an appropriate multidisciplinary infrastructure (16). These results are encouraging, though their effectiveness varies, and therapy is not equally effective across all disease types due to disease aggressiveness and the limited range of therapeutic options (1, 2).

A common translational finding is that the route of delivery substantially affects efficacy and toxicity. With intravenous CAR T-cell infusion for CNS tumors, obstacles may be faced due to poor trafficking across the BBB and ineffective homing to infiltrative tumor beds (2, 4, 10). Locoregional administration, including spinal intrathecal infusion, focal ultrasound, and intraventricular and intratumoral delivery, has been highlighted as a means to circumvent the trafficking barrier, thereby increasing the effective concentration at the disease site while potentially reducing systemic exposure (5, 12, 16, 17). Reviews of current trials indicate that locoregional infusion strategies may enhance pharmacologic and biologic access to tumor cells and may be central to achieving consistent activity in pediatric CNS environments (1, 2, 17).

### Safety and adverse events

One of the important challenges in CAR T-cell therapy in pediatric brain tumors is safety, owing to CAR-related toxicities and intracranial inflammation, which can rapidly escalate in a confined space (2, 10, 15). Cytokine release syndrome (CRS), a systemic inflammatory syndrome due to immune activation and cytokine release, has been observed in CAR T-cell therapy and can also occur in CNS tumor CAR T-cell settings, for which the common management is supportive care and cytokine-directed therapies such as IL-6 blockade (2, 6, 18). In pediatric CNS CAR T-cell studies, neurotoxicity is the prominent adverse event, and GD2-CAR T-cell treatment has been associated with neurotoxic events, including encephalopathy, underscoring the need for careful monitoring of dosing and risk-mitigation protocols (1, 4). This phenomenon, termed tumor inflammation-associated neurotoxicity (TIAN), occurs due to CAR T-cell-mediated inflammation and was the dose-limiting toxicity that defined the maximum tolerated intravenous dose of 1 × 10^6^ cells/kg in the GD2-CAR T-cell trial. High-grade cytokine release syndrome (CRS) was the principal dose-limiting event following intravenous dosing, followed by neurotoxicity and CRS, which was reversible with supportive care, including dexamethasone and, for CRS, IL-6-receptor blockade (tocilizumab), and no treatment-related deaths occurred (15).

Antigen selection and target distribution are also central to safety. Targets with any meaningful expression in vital normal tissues raise the risk of ‘on-target, off-tumor’ injury. HER2 has historically been associated with such concerns, but controlled locoregional dosing and careful trial design supported a manageable safety profile, with no dose-limiting toxicities in the pediatric CNS context (12). Tolerability was shown along 253 infusions in 21 DIPG patients, with manageable adverse effects, and only 1 dose-limiting toxicity (intratumoral hemorrhage) was shown in a different study involving the ICV B7-H3 CAR T-cell therapy (5). Most adverse events in this trial graded by CTCAE v5.0 were grade 1–2, with the most common one being headache (81% of patients), nausea or vomiting (81%), fatigue (62%), and fever (57%); only a few number of patients developed grade 3 events, and the single dose-limiting toxicity (an intratumoral hemorrhage in a 3-year-old patient with progressive disease) was graded 4 and was eventually lethal due to underlying tumor progression rather than ongoing CAR T-related toxicity. In particular, no cases of immune-effector-cell-associated neurotoxicity syndrome were noticed in this cohort, and no patients needed treatment discontinuation due to CRS, which differentiates the safety profile of locoregional ICV delivery from systemic CAR T-cells administration (5). Moreover, no intra-Ommaya or intracerebrospinal fluid infections were seen among 307 doses delivered intracranially via catheterization at Seattle Children’s, suggesting procedural feasibility (16). Engineering controls such as suicide switches, affinity tuning, and logic-gated recognition systems are being used to grow specificity and avoid unintended activation (2, 3). These strategies are specifically vital in pediatrics, where long-term neurologic preservation is a major priority.

### Barriers to durable response

Although early tumor regression is achieved, attaining stable control is challenging. A major barrier is antigen heterogeneity, with variable target expression across pediatric brain tumors and a consequent risk of immune escape when only one antigen is targeted (2, 10, 14). This is especially important in pediatric CNS tumors, which consist of multiple molecular subclones and spatially distinct antigen landscapes, so a single-antigen CAR can be undermined over time, even when initially effective (2, 3, 10). Multi-antigen strategies, including dual CARs, tandem CARs, or pooled constructs, are increasingly focused on as practical solutions to reduce the potential for escape.

Another barrier is the immunosuppressive tumor microenvironment. Pediatric CNS tumors can recruit immunosuppressive myeloid populations, express inhibitory ligands, and secrete cytokines that limit T-cell activity, thereby decreasing persistence and increasing exhaustion (2, 4, 18). Interim analyses from IL13Rα2-CAR trials have highlighted that, while endogenous T-cell expansion in CSF was observed, CAR T-cell persistence in the tumor microenvironment remained limited, underscoring the need for engineering approaches to improve CAR T-cell fitness and resistance to immunosuppression (13).

Finally, the blood-brain barrier and the structure of the central nervous system create physical and pharmacologic barriers, making it harder for the therapy to reach tumor sites and to manage ongoing inflammation (2, 4, 10). A combination of improved targeting, better delivery, and smarter cellular engineering is required, since no single approach fully addresses heterogeneity, immunosuppression, or the risk of neuroinflammatory toxicity.

### Comparative analysis of trial eligibility criteria

A structured search of international trial registries identified 12 active, completed, or pending CAR T-cell trials enrolling pediatric patients with primary CNS tumors (**Table 2**). Trials spanned four countries (USA, Italy, UK, and Australia), with nine conducted at single centers and nine based in the United States, four of which were at Seattle Children’s Hospital alone.

Age eligibility varied across trials. Most enrollments allowed intake from 1 year of age, although five trials set higher minimum age limits: NCT03638167 with 15 years or older; NCT06946680 with 4 years or older; and ISRCTN15356845 with 2 years or older. The upper age limit ranged from 16 years (ISRCTN15356845) to 30 years (NCT05298995; NCT07087002), with the majority averaging 25–26 years. No trial provided an explicit published rationale for these age boundaries in its eligibility documentation.

Performance status thresholds were relatively consistent, with seven of twelve trials requiring a Lansky or Karnofsky score of ≥60%, NCT06946680 with > 70% , and NCT07087002 not stated. Three trials accepted lower thresholds: NCT04099797 and ACTRN12622000675729 permitted a score of ≥50%, and ISRCTN15356845 accepted ≥40% — a meaningfully more inclusive criterion that would allow enrollment of patients excluded from the majority of trials.

H3K27M mutation requirements differed substantially across GD2-targeting trials despite shared biological rationale. The trial from Australia (ACTRN12622000675729) required either an H3K27M mutation or loss of H3K27 trimethylation as an obligatory inclusion criterion. NCT04099797 had a more conditional approach: either GD2 expression or a positive H3K27M status, when GD2 testing was unavailable, was included in the study. The Italian GD2 trial (NCT05298995) did not include H3K27M positivity as an eligibility criterion. This heterogeneity means that a child with GD2-positive, H3K27M-wildtype DIPG would qualify for some GD2 trials but not others — a distinction not apparent from trial titles or summaries.

Route of administration varied considerably: four trials used ICV delivery exclusively, four used intravenous infusion only, and three employed sequential or combined IV-to-ICV approaches. Among all GD2 trials, there was a different delivery strategy used in each of the three, ruling out pharmacokinetic or efficacy differences across them.

Prior therapy requirements divided all trials evenly: seven did not require prior therapy beyond standard radiotherapy, whereas five required documented progression after each added treatment line. In a disease where median survival is under 12 months, this requirement materially delays access for some patients to experimental therapy.

## Discussion

### Translational disparity between preclinical potency and clinical outcomes

The evidence for CAR T-cell therapy for pediatric brain tumors is promising yet clinically complex. Preclinical studies support the feasibility, particularly for antigens such as B7-H3 and GD2, in which tumor control has been observed in relevant models (4, 8). However, early clinical data show heterogeneous outcomes with modest response rates, reflecting the challenges posed by CNS tumors and the realities of cell therapy in pediatric neuro-oncology populations (1, 12). The gap between preclinical potency and clinical consistency is likely attributable to interacting factors, including antigen heterogeneity, adaptive resistance, limited trafficking and persistence, and a suppressive TME that is difficult to remodel with CAR T-cells alone (2, 10, 17).

### Delivery strategy as a modifiable translational lever

One of the most actionable translational levers is the delivery route (**Table 3**). Locoregional approaches, including intraventricular and intratumoral infusions, have both biological rationale and clinical experience, as they improve CNS drug exposure while minimizing reliance on systemic transport across the blood-brain barrier (5, 12, 16). In a pediatric setting, these approaches can also allow for titration of dose and scheduling to balance out the neurotoxicity risk. The B7-H3 ICV phase 1 trial in DIPG exemplifies this approach, demonstrating that multi-year, repeated ICV dosing is both feasible and tolerable, with preliminary efficacy signals (5). Nevertheless, locoregional delivery is not a definitive solution; it requires neurosurgical expertise, carries procedural risks, and may still encounter infiltrative disease beyond the infusion field (2, 16). Commonly used animal models contribute significantly to a disconnect seen between preclinical success and clinical outcomes. Xenograft models demonstrate initial antitumor activity but lack an immune system, thus showing an inaccurate representation of the complex tumor microenvironment in pediatric CNS tumors. Significantly superior models like syngeneic orthotopic glioma models and humanized or patient-derived orthotopic xenograft systems exist, which show better preservation of the tumor architecture and immune system. These models are more realistic in analyzing CAR T-cell trafficking and its neurotoxicity in the CNS. Integration of these systems early on, can improve the reliability of preclinical findings and bridge the disconnect seen between preclinical studies and clinical outcomes. .

**Table 3:** Delivery strategies for CAR t-cell therapy in pediatric CNS tumors [Ronsley et al., 2024 (1); Vitanza et al., 2025 (5), 2021 (12), 2023 (16); Wang et al., 2023 (13); Rao et al., 2022 (2); Yaacoub et al., 2025 (3); Timpanaro et al., 2023 (17); Ferreras et al., 2021 (18)].

Delivery route	Biological rationale	Advantages	Limitations
Intravenous (Systemic)	Standard CAR T-cell administration relies on trafficking across the BBB	Less invasive; scalable; no neurosurgical requirement	Limited CNS penetration; variable trafficking; systemic toxicity risk
Intratumoral	Direct delivery into the tumor mass	High local concentration; bypasses the BBB	Procedural risk, limited diffusion, not ideal for infiltrative disease
Intraventricular	Distribution through CSF pathways	Broader CNS exposure; dose titration possible	Requires neurosurgical access; procedural risk; may not reach all tumor compartments
Repeated Locoregional Dosing	Scheduled CNS-directed administration	Adjustable dosing; potential toxicity modulation	Logistical complexity; infection risk; requires monitoring infrastructure

BBB, blood-brain barrier; CAR T-cell, chimeric antigen receptor T-cell; CNS, central nervous system; CSF, cerebrospinal fluid.

### Engineering innovation: toward durable and context-aware CAR T-cell platforms

Engineering innovation can likely determine if CAR T-cell therapy becomes durable and scalable. To reduce escape and spatial heterogeneity, multi-antigen targeting and bispecific designs are increasingly being prioritized (2, 3). Logic-gated and context-dependent systems can improve specificity and reduce off-tumor risk, a pivotal consideration for targets that may have low-level expression in normal tissue (2). ‘Armored’ CAR T-cells, engineered for improved persistence and resistance to suppression, represent another major frontier, especially as the field acknowledges that the TME is an active adversary to sustained T-cell function (2, 4). Novel pan-tumor target strategies—such as targeting cancer-specific exons—and next-generation antigen discovery approaches that leverage IL-18R support and oncofetal antigens are also emerging avenues to broaden pediatric CAR T-cell applicability. The major next-generation engineering strategies currently under investigation are outlined in **Table 4**.

**Table 4:** Engineering strategies for CAR T-cell therapy in pediatric CNS tumors: mechanisms, intended benefits [Rao et al., 2022 (2); Yaacoub et al., 2025 (3); Ronsley et al., 2024 (1); Ferreras et al., 2021 (18); Thomas et al., 2021 (14)].

Strategy	Mechanism	Intended benefit
Bispecific CARs	Dual antigen recognition	Reduce escape
Tandem CAR constructs	Simultaneous multi-targeting	Address heterogeneity
synNotch systems	Context-dependent activation	Improve specificity
Armored CAR T-cells	Cytokine secretion/resistance to suppression	Enhance persistence
Suicide switches	Controlled elimination	Improve safety

CAR T-cell, chimeric antigen receptor T-cell; synNotch, synthetic Notch receptor system (logic-gated signaling platform).

### Neurotoxicity and safety architecture in pediatric CNS applications

Safety considerations remain paramount. Clinical experience with GD2- CAR T-cell therapy demonstrates that neurotoxicity can be dose-limiting and that brain inflammation carries higher risks than similar immune activation in systemic settings (1, 4). These high stakes call for incorporating controllability via suicide switches, affinity tuning, and rigorous monitoring pathways as a standard feature in pediatric CNS CAR T-cell development (2, 3). The ICV B7-H3 trial’s multi-year safety record across 253 infusions in 21 DIPG patients further underscores that, with adequate monitoring infrastructure, repeated intracranial CAR T-cell delivery can be made safe for children (5). Lastly, long-term follow-up is essential not only for survival but also for neurodevelopmental outcomes, as therapies that preserve life but impair neurologic function can impose substantial lifelong costs (1, 5).

### Pediatric CAR T-cell trials

Across pediatric CAR T-cell trials, the observed heterogeneity had meaningful implications for patient access, cross-trial comparisons, and the order of future trials. Much of this heterogeneity is institutionally driven, but some discrepancies, such as requirements for H3K27M or GD2 mutations, have a biological rationale.

The discrepancy with performance thresholds is perhaps the most clinically important finding. Children suffering from DIPG have progressive neurological deficits, which manifest as a reduction in the Lansky or Karnofsky score. In most trials, a common threshold of the Lansky or Karnofsky score ≥60% was used; however, some trials used ≥50% (19), and others ≥40% (20). This means a much sicker patient is enrolled in the trial than one requiring a score of ≥60%, introducing a confounder in cross-trial comparisons. For a disease with a median survival of under 12 months, even a marginal difference in this eligibility criterion can determine whether a sick child is fit for a clinical trial like this one. Future studies should focus on synchronizing this data to avoid comparisons in trial outcomes.

Similarly, some trials require an H3K27M mutation, while others require only a GD2-positive tumor. This has consequences as GD2 expression and H3K27M mutation status are correlated- in the sense that 80–90% of H3K27M-mutations express GD2 protein (15). On the contrary, not every GD2-positive tumor harbors the H3K27M mutation, meaning that a subset of patients will be excluded from these trials. Future trials should have more precise inclusion criteria for mutational status, with GD2 testing at enrollment, to avoid excluding participants who could benefit from the trials.

Yet another discrepancy observed is the geographic concentration: seven of the twelve trials were conducted in the USA. Out of these, four trials are at a single center. This compounds inequities, with families outside these academic centers having less access to these trials, resulting in a higher disease burden. Multi-site study designs should be adopted to prevent this discrepancy.

Finally, enrollment delays pose a real risk. Patients enrolled in multiple trials received no guidance. As these trials approach Phase 2, they should focus more on these limitations by using standardized GD2 expression testing, setting equalized performance thresholds, and enrolling multiple centers, as a practical goal for this field of study.

### Clinical evidence gaps and future trial design priorities

The current clinical data are limited by small cohorts, differing eligibility criteria, varying tumor subtypes, prior therapy, and modes of delivery. In the future, multi-institutional collaboration and clinical trials are necessary to determine which patient groups would benefit most, identify the optimal timing of therapy administration (e.g., earlier lines versus end-stage disease), and integrate CAR T-cell therapy into multimodal treatment paradigms (1, 2, 17). Findings from IL13Rα2-CAR T-cell trials highlight the importance of longitudinal analyses of CSF, blood, and tumor using single-cell approaches to understand and refine cellular immunotherapies for pediatric brain tumors (13). Comprehensive literature reviews and trial registries of current North American trials further delineate evidence gaps and identify areas for investigation (1, 17).

## Limitations

This review has several limitations in its narrative design. Narrative reviews are susceptible to a positive publication bias, where a favorable outcome is more likely to be published and insignificant findings do not proceed due to their lack of efficacy, therefore being underrepresented. This can potentially inflate the value of certain antigen targets or delivery methods. Additionally, trials that were withdrawn were not added to the comparative analysis and these trials may have some information on safety and feasibility. The trial registry search (April 2025) may not include studies registered in non-English-language or regional registries other than ClinicalTrials.gov, ISRCTN, and ANZCTR. Formal risk-of-bias tools were not applied and meta-analysis could not be conducted due to the heterogeneity in studies; findings should therefore be interpreted as generating a hypothesis. Finally, results published after the 2025 search cutoff may alter the conclusions drawn here.

## Conclusion

In conclusion, CAR T-cell therapy shows significant potential to transform pediatric neuro-oncology, particularly for tumors with poor outcomes under current standards of care. Significant preclinical efficacy has been demonstrated for multiple tumor-associated antigens, mainly GD2 and B7-H3, and early clinical trials—including the phase 1 GD2-CAR T-cell trial in diffuse midline gliomas and the ICV B7-H3 trial in DIPG—provide promising, if preliminary, evidence of activity in select patients (1, 4, 5). HER2- and IL13Rα2-directed locoregional trials have further advanced the clinical feasibility of intracranial CAR T-cell delivery in children (12, 13). Nevertheless, long-lasting benefit is limited by antigen heterogeneity, trafficking challenges, immune suppression within the CNS tumor microenvironment, and the risk of neurotoxicity. Future developments will mainly depend on multi-antigen and logic-gated CAR designs, as well as ideas that enhance persistence and minimize immune suppression (2, 3). Carefully planned pediatric trials will be equally important, incorporating rigorous safety controls and long-term neurodevelopmental follow-ups to ensure both efficacy and safety. This review additionally presents a structured comparative analysis of eligibility criteria across active CAR T-cell trials in pediatric CNS tumors, identifying meaningful heterogeneity in age thresholds, performance status cutoffs, molecular requirements, and delivery routes that warrants standardization in future multi-site trial design.

## References

[1] Ronsley R Bertrand KC Song EZ Timpanaro A Choe M Tlais D . CAR T-cell therapy for pediatric central nervous system tumors: a review of the literature and current North American trials. Cancer Metastasis Rev. (2024) 43:1205–16. doi: 10.1007/s10555-024-10208-4 39251462 PMC11554695

[2] Rao P Furst L Meyran D Mayoh C Neeson PJ Terry R . Advances in CAR T-cell immunotherapy for paediatric brain tumours. Front Oncol. (2022) 12:873722. doi: 10.3389/fonc.2022.873722 36505819 PMC9727400

[3] Yaacoub R Bahmad HF Dagher A Moussa S Kobeissy F Salam I . CAR T-cell therapy in brain Malignancies: obstacles in the face of cellular trafficking and persistence. Front Immunol. (2025) 16:1596499. doi: 10.3389/fimmu.2025.1596499 40612954 PMC12222291

[4] Mount CW Majzner RG Sundaresh S Arnold EP Kadapakkam M Haile S . Potent antitumor efficacy of anti-GD2 CAR T-cells in H3-K27M+ diffuse midline gliomas. Nat Med. (2018) 24:572–9. doi: 10.1038/s41591-018-0006-x 29662203 PMC6214371

[5] Vitanza NA Ronsley R Rawlings-Rhea SD Wilson AL Gustafson JA Goldstein HE . Intracerebroventricular B7-H3-targeting CAR T-cells for diffuse intrinsic pontine glioma: a phase 1 trial. Nat Med. (2025) 31:861–8. doi: 10.1038/s41591-024-03451-3 39775044 PMC11922736

[6] Huang Z Huang Y Lin J Liao W Xiao D Wang Q . Emerging trends in gene-modified-based chimeric antigen receptor–engineered T-cellular therapy for Malignant tumors: the lesson from leukemia to pediatric brain tumors. J Chin Med Assoc. (2020) 83:704–11. doi: 10.1097/JCMA.0000000000000358 32773642 PMC12991742

[7] Patterson JD Henson JC Breese RO Bielamowicz KJ Rodriguez A . CAR T-cell therapy for pediatric brain tumors. Front Oncol. (2020) 10:1582. doi: 10.3389/fonc.2020.01582 32903405 PMC7435009

[8] Majzner RG Theruvath JL Nellan A Heitzeneder S Cui Y Mount CW . CAR T-cells targeting B7-H3, a pan-cancer antigen, demonstrate potent preclinical activity against pediatric solid tumors and brain tumors. Clin Cancer Res. (2019) 25:2560–74. doi: 10.1158/1078-0432.CCR-18-0432 30655315 PMC8456711

[9] Haydar D Houke H Chiang J Yi Z Odé Z Caldwell K . Cell-surface antigen profiling of pediatric brain tumors: B7-H3 is consistently expressed and can be targeted via local or systemic CAR T-cell delivery. Neuro Oncol. (2021) 23:999–1011. doi: 10.1093/neuonc/noaa278 33320196 PMC8168826

[10] Burns D Gwynne WS Suk Y Custers S Chaudhry N Venugopal C . The road to CAR T-cell therapies for pediatric CNS tumors: obstacles and new avenues. Front Oncol. (2022) 12:815726. doi: 10.3389/fonc.2022.815726 35155252 PMC8829546

[11] Wu WT Lin WY Chen YW Lin CF Wang HH Wu SH . New era of immunotherapy in pediatric brain tumors: chimeric antigen receptor T-cell therapy. Int J Mol Sci. (2021) 22:2404. doi: 10.3390/ijms22052404 33673696 PMC7957810

[12] Vitanza NA Johnson AJ Wilson AL Brown C Yokoyama JK Künkele A . Locoregional infusion of HER2-specific CAR T-cells in children and young adults with recurrent or refractory CNS tumors: an interim analysis. Nat Med. (2021) 27:1544–52. doi: 10.1038/s41591-021-01404-8 34253928

[13] Wang L Oill A Blanchard M Wu M Hibbard J Sepulveda S . Expansion of endogenous T cells in CSF of pediatric CNS tumor patients undergoing locoregional delivery of IL13Rα2-targeting CAR T-cells: an interim analysis. (2023). doi: 10.21203/rs.3.rs-3454977/v1. Preprint.

[14] Thomas P Galopin N Bonérandi E Clémenceau B Fougeray S Birklé S . CAR T cell therapy’s potential for pediatric brain tumors. Cancers (Basel). (2021) 13:5445. doi: 10.3390/cancers13215445 34771608 PMC8582542

[15] Majzner RG Ramakrishna S Yeom KW Patel S Chinnasamy H Schultz LM . GD2-CAR T cell therapy for H3K27M-mutated diffuse midline gliomas. Nature. (2022) 603:934–41. doi: 10.1038/s41586-022-04489-4 35130560 PMC8967714

[16] Vitanza NA Ronsley R Choe M Henson C Breedt M Barrios-Anderson A . Locoregional CAR T cells for children with CNS tumors: clinical procedure and catheter safety. Neoplasia. (2023) 36:100870. doi: 10.1016/j.neo.2022.100870 36599192 PMC9823206

[17] Timpanaro A Song EZ Choe M Crotty EE Foster JB Jung S . The peculiar challenge of bringing CAR T cells into the brain: perspectives in the clinical application to the treatment of pediatric central nervous system tumors. Front Immunol. (2023) 14:1142597. doi: 10.3389/fimmu.2023.1142597 37025994 PMC10072260

[18] Ferreras C Fernández L Clares-Villa L Ibáñez-Navarro M Martín-Cortázar C Esteban-Rodríguez I . Facing CAR T cell challenges on the deadliest paediatric brain tumours. Cells. (2021) 10:2940. doi: 10.3390/cells10112940 34831165 PMC8616287

[19] Mahdi J . C7R-GD2.CAR T cells for patients with GD2-expressing brain tumors (GAIL-B). In: Clinicaltrials.Gov (2025). Available online at: https://clinicaltrials.gov/study/NCT04099797?term=NCT04099797&viewType=Card&rank=1 (Accessed April 7, 2026).

[20] A CAR T study for paediatric-type diffuse high-grade gliomas, including diffuse midline glioma. In: Isrctn.Com. Available online at: https://www.isrctn.com/ISRCTN15356845?q=ISRCTN15356845&filters=&sort=&offset=1&totalResults=1&page=1&pageSize=10 (Accessed April 7, 2026).

